# Extraction/transportation of Co2+ metal ions with bis(2-ethylhexyl) phosphate dissolved in kerosene using a multidropped liquid membrane technique

**DOI:** 10.55730/1300-0527.3619

**Published:** 2023-08-13

**Authors:** Volkan DEMİREL, Ramazan DONAT

**Affiliations:** Department of Chemistry, Faculty of Science, Pamukkale University, Denizli, Turkiye

**Keywords:** Cobalt, D2EHPA, transport, membrane, multidroplet liquid membrane system

## Abstract

The transport properties of Co^2+^ ions from the aqueous donor phase to aqueous acceptor phase with the recently developed multidroplet liquid membrane (MDLM) extraction system were studied. This system serves as a continuous process for the transportation of ions and requires fewer reagents for starting and conducting the procedure. Moreover, the procedure results in fewer waste chemicals and mixtures in comparison to traditional extraction methods. During extraction, bis(2-ethylhexyl) phosphate (D2EHPA) was used as a carrier material and 5% potassium thiocyanate (KSCN) solution was used to obtain a colored complex for UV-Vis detection. By means of several experiments, the optimum D2EHPA concentration, pH range for both donor and acceptor phases, and temperature range effect on transport kinetics were investigated. In the extraction of cobalt ions with the MDLM system, the activation energy was calculated as *E*_a_ = 13.80 kcal mol^−1^, and it was found that the extraction was chemically controlled since it was greater than 10 kcal mol^−1^.

## 1. Introduction

With the rapid development of industry, the prevalence of electrical household tools, changes in transportation, and the increasing volume of chemical usage, especially in chemical, agricultural, and construction areas, have resulted in the emergence of various types of contaminants in the environment [1–5]. The most prevalent group of pollutants that hinders nature’s ability to regenerate is heavy metal pollution [6,7].

Environmental protection studies have mostly concentrated on heavy metals such as lead, cadmium, and mercury, but other elements also need to be taken into consideration due to the possibility of significant soil loading [1]. One element that is found in soil, water, and air environments in quantities greater than trace values is cobalt, which also garners special attention [8].

Cobalt has very similar properties to iron and nickel, and it is found naturally in air, water, soil, and living organisms. Cobalt has an atomic number of 27 and only one stable isotope with an atomic weight of 58.93 g mol^−1^ [9]. Although cobalt compounds were used as coloring agents in glass around 4000 years ago, up until 1914 cobalt was not produced as a pure metal. However, it was isolated and recognized much earlier; in 1735, Swedish chemist Brandt achieved cobalt purification [10].

Cobalt is used in many industrial areas, which include high-quality steel and alloy production. Some kinds of coloring agents, paint-drying agents, varnishes, enamels, and inks are produced with cobalt compounds [11]. Aside from industrial cobalt use, B_12_, or cyanocobalamin, is a biochemically essential cobalt compound for the healthy lives of animals and humans [12]. Food, water, air, or skin absorption are the main routes through which heavy metals enter the body [13].

The effluents from many industries, including textiles, leather, tanneries, electroplating, galvanizing, pigment and dye production, metallurgical and paint production, and other metal-processing and refining operations, can contain high levels of toxic metals [13]. Food is the primary source of cobalt intake for humans and 6 μg of B_12_ vitamin is the recommended intake value for the average person to maintain a healthy metabolism [14].

Some metals such as copper, zinc, cobalt, and iron are essential for human health, but above certain concentrations in the body, they can have toxic effects. The reason is most probably that they form complexes with protein molecules, rendering them inactive, such as in the case of B enzyme inactivation [15].

Excess amounts of cobalt in the human body can lead to intercellular hypoxia, asthma, and goiter [16]. Radioactive exposure to cobalt has serious health effects. In the case of a human interacting with radioactive cobalt, gamma rays damage cells in human tissues. Gamma rays can enter the entire body without physically interacting with radioactive cobalt. Because of the importance of removing cobalt from wastewater, numerous methods have been researched, including adsorption, biosorption precipitation, ion exchange, solvent extraction, and nanofiltration. However, none of these processes are practical or economical. Therefore, research based on the method of liquid membrane separation has gained importance [17]. Extraction systems based on liquid membranes include an immiscible liquid phase that acts as a semipermeable barrier between two other fluid parts; one of them provides (feeds) the solution, and the other receives (accepts) the solution [18]. Research done on liquid membranes involving both analytical and industrial applications has clearly proven that these techniques have many advantages [19]. Because they are straightforward and modular, are simple to scale up, and have low energy requirements, liquid membrane operations are perfect for industrial manufacturing. They are extremely energy-efficient and have little influence on the environment [20].

These techniques have three main configurations: bulk liquid membranes (BLMs), emulsion liquid membranes (ELMs), and supported liquid membranes (SLMs). Each configuration has some advantages and disadvantages derived from the nature of each system. For example, stability is the biggest problem for SLMs. Many factors affect the stability of this type of configuration, but the main impacts arise from liquid membrane elution, polymeric support stability, and possible emulsion formation in the liquid phase [19]. Emulsion stability is the primary problem with ELMs. In order to break the emulsion and reformulate it, the internal phase must be removed. The emulsion must be designed to survive the shear produced by mixing during the extraction. This necessitates a further step in the process as well as more energy inputs [21].

Due to the stability constraints of ELMs and SLMs, in recent years many researchers have started to develop alternative BLMs or bulk water-immiscible LMs [20]. The method used in this research entails a relatively new system that resembles the BLM technique and has many advantages in terms of both required materials and practicality. This method is called the multidropped liquid membrane (MDLM) approach. It is very efficient in terms of membrane capacity, separation factor, and ease of set-up.

The extraction of Co^2+^ ions was investigated in this study using the MDLM system. Kerosene was used for the organic phase and different solution properties were examined to explore the most effective extraction parameters.

## 2. Chemicals and experimental methods

### 2.1. Chemicals and solutions

Kerosene was purchased from TÜPRAŞ A.Ş. (İzmit, Türkiye); cobalt(II) nitrate, potassium thiocyanate (KSCN), nitric acid, sodium, and carbonate were obtained from Merck (Darmstadt, Germany); and bis(2-ethylhexyl) phosphate (D2EHPA) was purchased from Sigma-Aldrich (St. Louis, MO, USA).

A 250 mg L^−1^ stock solution of Co^2+^ was prepared by dissolving 0.3087 g of Co(NO_3_)_2_ in water. Co^2+^ solutions of lower concentration were prepared via dilution of the stock solution while 0.061, 0.045, 0.030, 0.023, and 0.015 mol L^−1^ D2EHPA solutions were prepared in kerosene. KSCN (12.5 g) was dissolved in 50% water and 50% ethanol by weight in order to prepare 250 mL of a 5% KSCN solution. This solution was used for spectrophotometric measurements of cobalt ions in the donor and acceptor phases.

### 2.2. Experimental procedure

**Figure 1:**
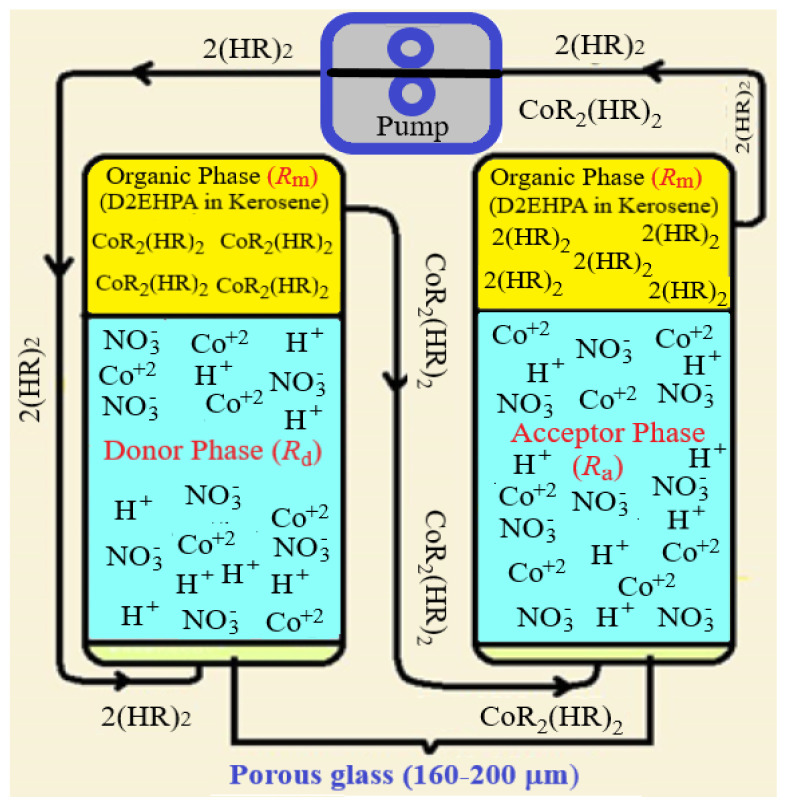
Measurement set schematic for flowing MDLM system.

In the experimental protocol, the MDLM method, which was developed at Pamukkale University, was used. A schematic diagram of the system is shown in Figure 1. In this system, one of the reactors includes a feed solution (heavy metal ions in water) at the bottom and an organic phase with a carrying agent at the top. There are two phases in both reactors due to the polarity characteristics of the solvents. Low-density organic phases are preferred in MDLM systems. The circulation of the organic base is carried out by a peristaltic pump, and in a closed pressure system, this phase continuously passes each water-based solution as many droplets. During the passage, chemical interactions occur, and due to the different pH levels of the solutions, metal ions are transported from the organic phase to the water phase in the acceptor phase, while in the donor phase, the opposite occurs.

The temperature of the reactors is fixed with water circulation around them, and for this purpose, a thermostat is used as a creosote device. A peristaltic pump is installed in the system to control the passage rate of the organic phase over the system. After the system is set up, the pump is started and samples are taken from the transmitter and receiver phases at 10-min intervals by means of a valve. The samples are then combined with a complexing agent to obtain a colored solution, and by UV-Vis spectrometer analysis, the absorbance of the solutions is recorded.

**Figure 2:**
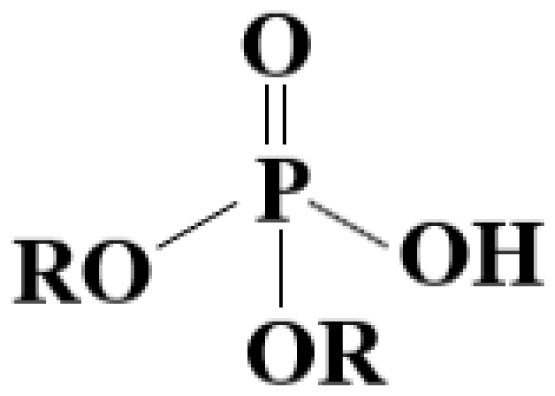
Chemical structure of D2EHPA.

In this study with the MDLM system, the weakly acidic cation exchanger D2EHPA was used as a reactive leaching agent and kerosene as an organic solvent. The chemical structure of D2EHPA is shown in Figure 2.

**Figure 3:**
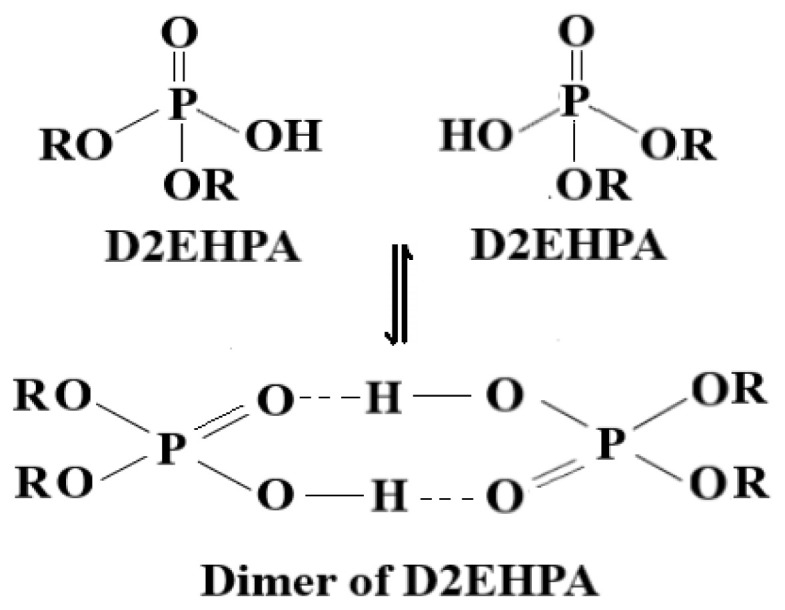
D2EHPA dimer structure in nonpolar kerosene.

**Figure 4:**
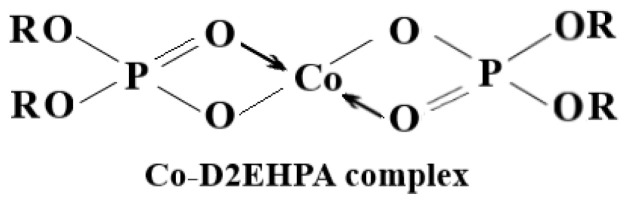
Cobalt ion complex formed by D2EHPA.

D2EHPA can be found as a dimer in a variety of organic solvents (hexane, isooctane, chloroform, kerosene, heptane, and toluene) [22–25]. D2EHPA is maintained as a dimer in nonpolar solvents like benzene, CCl_4_, and kerosene through hydrogen bonding (Figure 3). However, when heavy metal ions in the aqueous phase and D2EHPA in the organic phase come into contact, the carrier agent exhibits an affinity for the heavy metal ions in the aqueous phase and forms complexes with them (Figure 4). Figure 4 shows the complex formation of the carrier ligand D2EHPA with dimer formation in kerosene and with cobalt metal ions.

**Figure 5:**
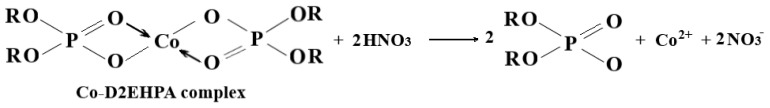
The process by which an uncharged compound between Co^2+^ and D2EHPA forms.

While the organic phase passes through the donor phase, D2EHPA reacts with Co^2+^ ions, and this reaction gives the Co-D2EHPA complex. The organic phase carries the Co-D2EHPA complex from the donor phase reactor to the acceptor phase reactor, and when droplets containing Co-D2EHPA pass through the acceptor phase, which is a medium containing nitric acid, Co^2+^ ions are separated from the carrier agent and stay in the acceptor phase. This elution procedure uses a dissociation reaction between CoR_2_(HR)_2_ and H^+^ at the organic/acceptor interface, and this part of the reaction is the rate-controlling step (Figure 5).

A Shimadzu UV-1201 V spectrophotometer (Shimadzu Corporation, Kyoto, Japan) was used to determine the absorbance of the samples obtained during the experiments. The reactors were equipped with a Labo SM3 type creosote instrument (Labo, İstanbul, Türkiye) to keep the system temperature in a fixed range. For the purpose of controlling organic phase circulation, the Longer Pump BT300-2J peristatic pump model (Longer Precision Pump Co., Ltd., Hebei, China) was used. For pH measurements, the WTW pH 7110 model pH-meter (Xylem Analytics, Weilheim, Germany) was utilized.

### 2.3. Data analysis

The main factor influencing the development of the ionic concentration of Co^2+^ in the membrane phase is material balance. Practical effects make reduced concentrations of Co^2+^ in the donor (*R*_d_), organic (*R*_m_), and acceptor phases (*R*_a_) dimensionless (*R*_d_ = Cdt/C_o_, *R*_m_ = C_mt_/C_o_, and *R*_a_ = C_at_/C_o_, the sum of *R*_d_ + *R*_m_+ *R*_a_ unity). After the determination of *R*_d_, *R*_m_, and *R*_a_ values according to the kinetic scheme, the results showed that the kinetic laws of two consecutive irreversible first-order reactions fit the Co^2+^ ion transport reaction mechanism [26].


(1) $$R_{\text{d}}\overset{k_{1}}{\to }R_{\text{m}}\overset{k_{2}}{\to }R_{\text{a}}$$

Here, *R*_d,_ *R*_m_, and *R*_a_ are Co^2+^ concentrations in the donor, membrane, and acceptor phases, respectively. The pseudo-first-order apparent rate constants of the extraction and reextraction are *k*_1_ and *k*_2_.


(2) $$\frac{dR_{d}}{dt}=-k_{1}R_{d}\equiv J_{d/m}$$

(3) $$\frac{dR_{m}}{dt}=k_{1}R_{d}-k_{2}R_{m}$$

(4) $$\frac{dR_{da}}{dt}=k_{2}R_{m}\equiv J_{m/a}$$

Here, *J*_d/m_ and *J*_m/a_ represent the flux rate from the donor phase to the organic phase and from the organic phase to the acceptor phase, respectively. Provided that *k*_1_ ≠ *k*_2_, integration of Eqs. (2)–(4) gives the following expressions [27]:


(5) $$R_{d}=\text{exp}(-k_{1}t)$$

(6) $$R_{m}=\frac{k_{1}}{k_{2}-k_{1}}[\text{exp}(-k_{1}t)-\text{exp}(-k_{2}t)]$$

(7) $$R_{a}=1-\frac{1}{k_{2}-k_{1}}[k_{2}\;\text{exp}(-k_{1}t)-k_{1}\;\text{exp}(-k_{2}t)]$$

Here, *k*_1_ and *k*_2_ are the rate constants of extraction and reextraction, respectively. These equations prove that the time dependence of *R*_d_ is monoexponentially large and that the time dependence of both *R*_m_ and *R*_a_ is biexponential. The maximum value of *R*_m_ is seen at the time obtained from the following:


(8) $$\begin{array}{l} \text{d}R_{m}/\text{dt}=0\\ t_{m}^{max}=\frac{\text{ln }(\frac{k_{1}}{k_{2}})}{k_{1}-k_{2}} \end{array}$$

The maximum *R*_m_ value will then be:


(8) $$R_{m}^{max}={\left(\frac{k_{1}}{k_{2}}\right)}^{\frac{k_{2}}{k_{1}-k_{2}}}$$

A combination of Eqs. (8) and (9) can be obtained as in the following equation:


(10) $$k_{2}=\frac{\text{ln}\;\left(\frac{1}{R_{m}^{max}}\right)}{t_{max}}$$

First-order time differentiation of Eqs. (5)–(7) provides the final form of the flux equations [26].


(11) $$\frac{dR_{d}}{dt}=-k_{1}exp\;(-k_{1}t)$$

(12) $$\frac{dR_{m}}{dt}=\frac{k_{1}}{k_{2}-k_{1}}[\text{exp}(-k_{1}t)-exp(-k_{2}t)]$$

(13) $$\frac{dR_{a}}{dt}=\frac{k_{1}k_{2}}{k_{2}-k_{1}}[exp(-k_{1}t)-exp(-k_{2}t)]$$

In order to obtain maximum flux, the *t*_max_ given for Eq. (8) is substituted in Eqs. (11)–(13) [26,27].


(14) $${\left[\frac{dR_{d}}{dt}\right]}_{max}=-k_{1}{\left(\frac{k_{1}}{k_{2}}\right)}^{\frac{k_{1}}{k_{1}-k_{2}}}=J_{d}^{max}$$

(15) $${\left[\frac{dR_{dm}}{dt}\right]}_{max}=0$$

(16) $${\left[\frac{dR_{a}}{dt}\right]}_{max}=k_{2}{\left(\frac{k_{1}}{k_{2}}\right)}^{\frac{k_{2}}{k_{1}-k_{2}}}=J_{a}^{max}$$

(17) $$-{\left[\frac{dR_{d}}{dt}\right]}_{max}=+{\left[\frac{dR_{d}}{dt}\right]}_{max}\Rightarrow -J_{a}^{max}=+J_{a}^{max}$$

*k*_1_ and *k*_2_ can be acquired by putting experimentally obtained data into Eqs. (5)–(7). According to the experimental findings, *R*_d_ scales down exponentially with time, whereas *R*_a_ increases simultaneously. *R*_m_ showed its maximum value at intermediate times. Concentration variations of *R*_d_, *R*_m_, and *R*_a_ with respect to time in the experimental setup are given in Figure 1. The SigmaPlot software program was used to calculate actual numerical values. The Arrhenius equation was used to calculate activation energy values via *k*_1_ and *k*_2_ at different temperatures.


(18) $$\text{ln}(J)=\text{ln}(A)-\frac{E_{a}}{R}\left(\frac{1}{T}\right)$$

Transportation process kinetics over the MDLM system can be defined as a first-order reaction for metal ion concentrations:


(19) $$ln\;\left(\frac{C_{0}}{C_{e}}\right)=kt$$

Here, *C*_e_ is the metal ion concentration in the feed phase at a specified time, *C*_o_ is the starting concentration of the metal ions in the donor phase, *k* is the rate constant (min^−1^), and *t* is time (min). The *k* values were calculated from the plots of ln (*C*_o_/*C*_e_) vs. time.

For the calculation of the transported metal ion percentage, Eq. (20) can be used.


(20) $$E\%=\frac{{\left[Co(II)\right]}_{strip(t)}}{{\left[Co(II)\right]}_{\left(o\right)}}\times 100$$

Here, [Co(II)]_strip(t)_ is the metal concentration in the stripping phase at any given time.

### 2.4. General study of the system with different parameters

In order to carry out the extraction of Co^2+^ via MDLM, D2EHPA reagent in kerosene was used as a carrier agent and 100 mg L^−1^ of Co^2+^ was used as the donor phase. The effects of temperature, pH, and carrier agent concentration were investigated with a series of experiments.

## 3. Results and discussion

### 3.1. Effect of carrier agent concentration

**Figure 6:**
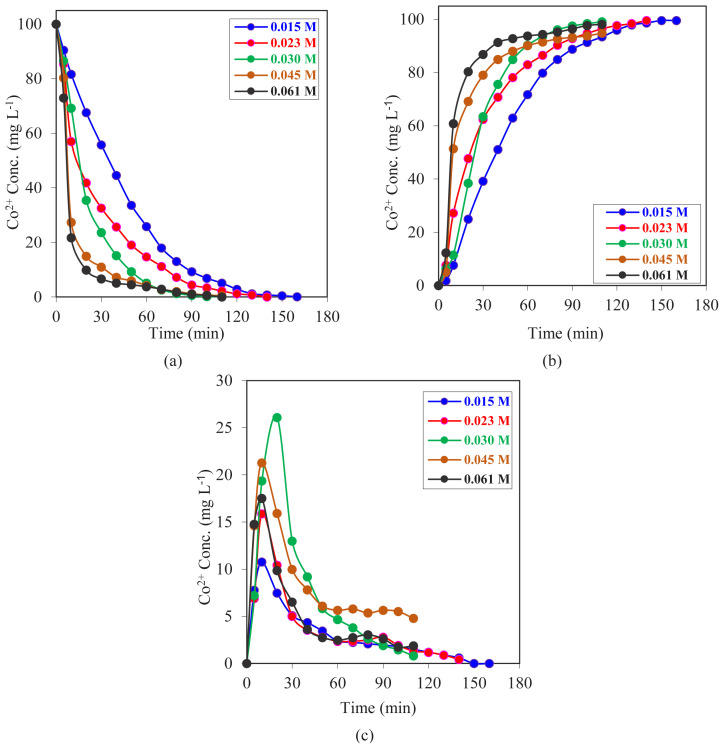
Concentrations of Co^2+^ ions versus time graphs for MDLM extraction studies of (a) donor, (b) acceptor, and (c) organic phase at different concentrations of carrier agent D2EHPA in kerosene (0.015–0.061 mol L^−1^).

In this study, a total of 100 mL of organic phase was used for each experiment. A series of experiments were carried out with the purpose of examining the effects of different amounts of carrier agent concentration (0.061, 0.045, 0.030, 0.023, and 0.015 mol L^−1^) on the extraction characteristics of Co^2+^ ions with the MDLM system, and then the results were interpreted. The data obtained from each experiment set are summarized in Figure 6. It was observed that increasing the carrier agent concentration in the organic phase resulted in a higher cobalt transportation rate from the donor phase to the organic phase.

The transport efficiency of Co^2+^ ions from the donor phase to the acceptor phase is greater than 94.79% according to Eq. (20). Figure 6 summarizes all collected data for Co^2+^ ion concentrations in each phase as a function of time. The highest transportation value was seen at a concentration of 0.023 mol L^−1^ of carrier agent. At this reagent concentration, a decrease in Co^2+^ ion concentration in the donor phase was followed by a consistent increase in Co^2+^ in the acceptor phase. The carrier agent concentration of 0.015 mol L^−1^ had the same trend, but the reaction time was slightly higher than that of the experiment that entailed a 0.023 mol L^−1^ carrier agent concentration. Other experiments with higher carrier agent concentrations showed faster transport of Co^2+^ ions from the donor phase to the organic phase, the transportation from the organic phase to the acceptor phase was slower, and an accumulation of carrier-metal ion complexes in the organic phase was observed. This phenomenon can be connected to several effects. The most probable influential effect is the connection of the free ion to another empty carrier molecule after its release from the first carrier molecule it was connected to in the donor phase. Although the pH effect is dominant for metal ion release in the acceptor phase, excess amounts of carrier in the organic phase mean that bubbles can be an impediment to passing Co^2+^ ions from the organic phase to the acceptor phase by repeatedly forming complexes with metal ions.

When we compare the transportation durations of experiments that have different carrier agent concentrations, we see that durations vary between 110 and 160 min, and increasing the D2EHPA concentration reduces the durations up to a carrier concentration of 0.030 mol L^−1^. Above that concentration, the transportation time remains constant at 110 min. Thus, a concentration of 0.030 mol L^−1^ can be assumed to be the upper saturation level of the carrier agent. Similarly, Leon and Guzman [28] used a BLM system and D2EHPA as a carrier ligand in order to extract Co^2+^ ions from kerosene. These investigators observed that increasing the carrier agent concentration in the membrane phase caused a higher extraction constant but also a decrease in the stripping constant to a constant value. Moreover, they found that the counterion concentration was directly proportional to both the extraction and stripping constants. Another finding was that an increase in carrier or counterion concentration led to a significant increase in Co^2+^ maximum flux across the bulk liquid membrane [28].

**Figure 7:**
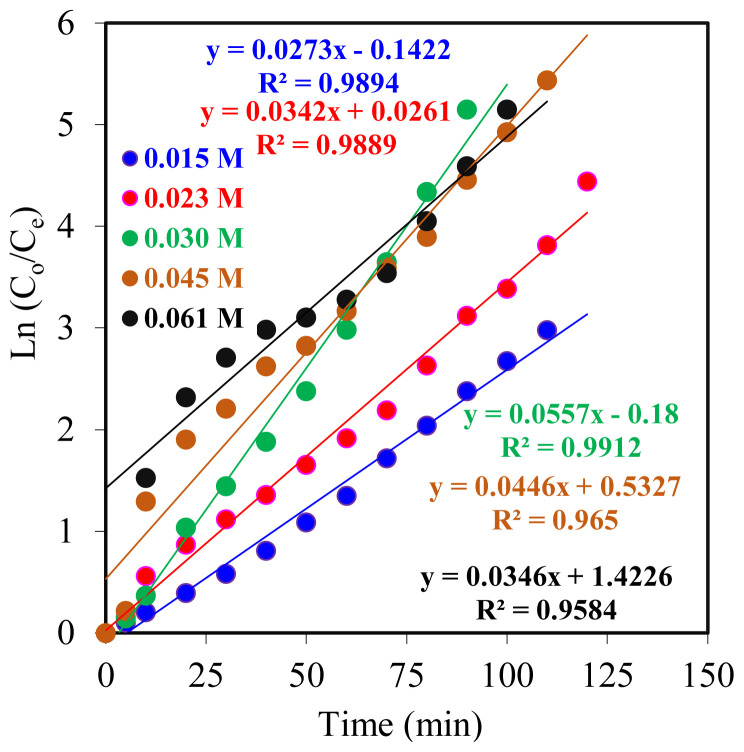
ln (*C*_o_/*C*_e_) versus time graphs for Co^2+^ ion MDLM extraction studies at different concentrations of carrier agent D2EHPA in kerosene (0.015–0.061 mol L^−1^).

By graphing the natural logarithm of the initial concentration of Co^2+^ ions from concentration (*C*_o_) to concentration (*C*_e_) over time, a kinetic curve can be obtained (Figure 7). Datasets for each individual experiment give a straight line, and this structure of the graph proves that the reactions are of the first order.

The least squares method was used to calculate *R*^2^ values, and all *R*^2^ values were found to be very close to 1.00, which shows that the data obtained from each experiment are highly consistent and there is a linear relationship between variables.

Table 1 summarizes the kinetic parameters of the effect of carrier agent concentration on the effectiveness of the MDLM system. *k*_1_ values increase from 0.015 mol L^−1^ to 0.030 mol L^−1^ but then decrease as the carrier agent concentration increases. At high concentrations of D2EHPA, the transportation rate is too high; therefore, a carrier agent concentration of 0.023 mol L^−1^ has the optimum kinetic parameter set with respect to other concentration sets.

### 3.2. Effect of temperature

**Figure 8:**
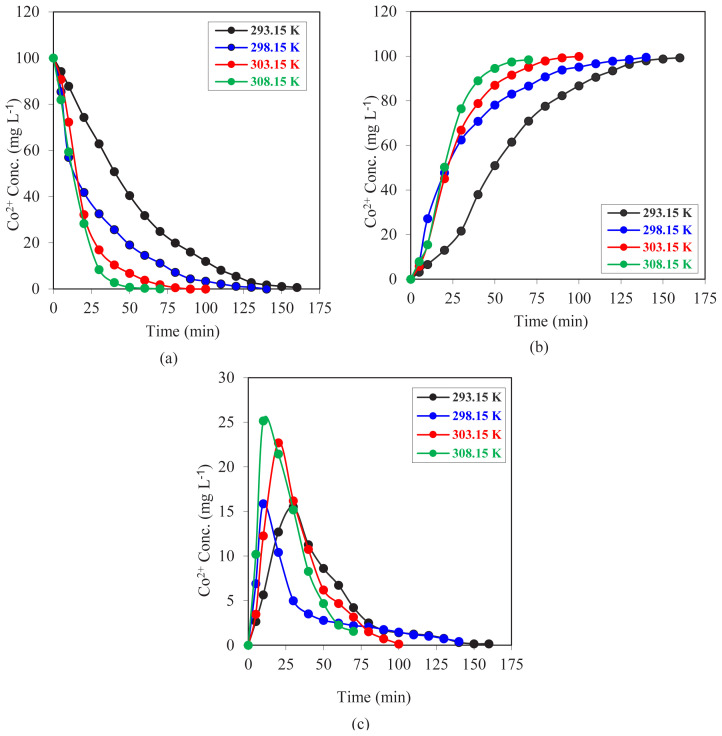
Concentrations of Co^2+^ ions versus time graphs for MDLM extraction studies of (a) donor, (b) acceptor, and (c) organic phase at different temperatures (293.15–308.15 K).

Experiments were performed at four different temperatures while holding other parameters constant to investigate the temperature effect on Co^2+^ extraction with the MDLM system. In these experiments, the donor, acceptor, and organic phases were 100 mL each; the carrier agent D2EHPA concentration was 0.023 mol L^−1^; and the pump speed was 20 rpm. Donor and acceptor phase pH values were 1.50 and 5.25, respectively. For four different temperatures (293.15, 298.15, 303.15, and 308.15 K), the change of the Co^2+^ ions’ concentration in the organic and aqueous phases over time was evaluated. Concentration versus time graphs for each phase were drawn and are shown in Figure 8. The highest transportation rate was achieved at 308.15 K according to the data collected. By lowering the temperature, the transportation rate was slowed. All experiments had transportation percentages greater than 98%, indicating that Co^2+^ ions were successfully transported from one reactor to another.

**Figure 9:**
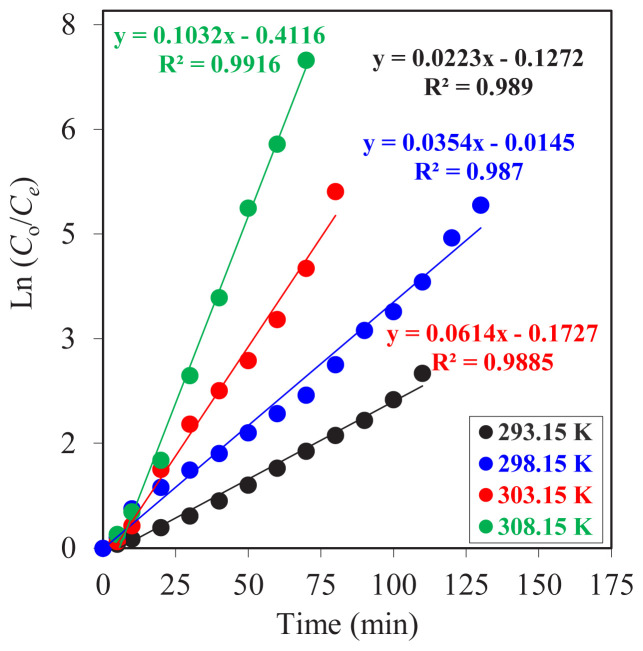
ln (*C*_o_/*C*_e_) versus time graphs for Co^2+^ ion MDLM extraction studies at different temperatures (293.15–308.15 K).

To evaluate kinetic curves and values while holding all other parameters constant and changing the reactor temperature level, the natural logarithm of the initial concentration of Co^2+^ ions (*C*_o_) to the concentration of Co^2+^ at the elapsed time (*C*_e_) versus time graph (Figure 9) was drawn. According to the obtained data, each individual experiment gave a straight line, and this proves that the reactions are of the first order. Increasing the temperature, the slope of each straight line was also raised. The *R*^2^ values, which were calculated with the least squares method, were very close to 1.00, and this shows the high consistency of the data collected from the experiments.

In Table 2, the kinetic parameters of the temperature effect on the effectiveness of the MDLM system are summarized. *k*_1_ values show an increasing trend from 293.15 K to 308.15 K. When we compare the *k*_2_ values, the highest value is obtained at a temperature of 308.15 K. At that temperature, the $k_{1}R_{m}^{max},J_{d}^{max}$ and $J_{a}^{max}$ values are also in very good ranges to facilitate effective transportation and regeneration of the carrier agent process.

**Figure 10:**
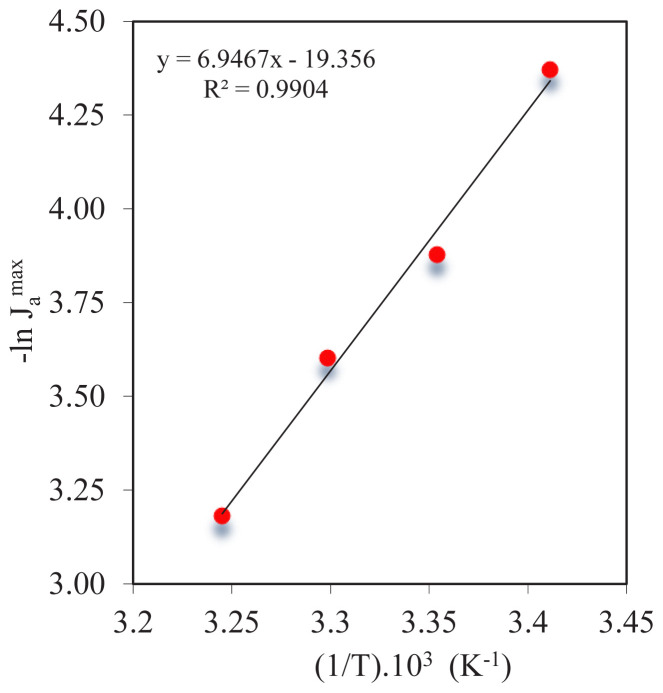
Graphical representation of the Arrhenius equation.

An essential element of the transport or extraction of metal ions from the MDLM system is activation energy. This was determined using Eq. (18), which describes the relation between transport rate and temperature via the MDLM technique. Experiments performed at different temperatures enabled the Arrhenius equation to be used to calculate the activation energy (Figure 10). The points that correspond to each individual value of the first Co^2+^ flux through the MDLM are connected by a straight line. The statistics show that the derived equation is significant; the *R*^2^ coefficient of correlation is equal to 0.9904 and this shows the significance of the relationship between temperature changes and $-\text{ln }(J_{a}^{max})$. With the slope of the straight line, the activation energy was calculated as 6.947, which is also statistically significant. Therefore, the activation energy is *E*_a_ = 13.80 kcal mol^−1^. Such a very low level of activation energy indicates that the transport of Co^2+^ ions to the organic phase containing D2EHPA via MDLM is kinetically controlled by the reaction of Co^2+^ with the carrier.

Evaluation of the activation energy provided in this research can be done by considering specific methodological problems defined by other researchers. First of all, the environments in which experimental studies are carried out cannot be compared. Secondly, it is difficult to compare extractors that differ greatly in terms of their chemical nature (acidic, neutral, and basic extractors) and the type of complexes they form (chelate complexes, ion pairs, or adducts). This line of reasoning is consistent with the presentations of many other authors, who hypothesized that activation energies of 10 kcal mol^−1^ or greater could indicate a transport-controlled chemical reaction [29,30].

### 3.3. Effect of pH level of donor phase

**Figure 11:**
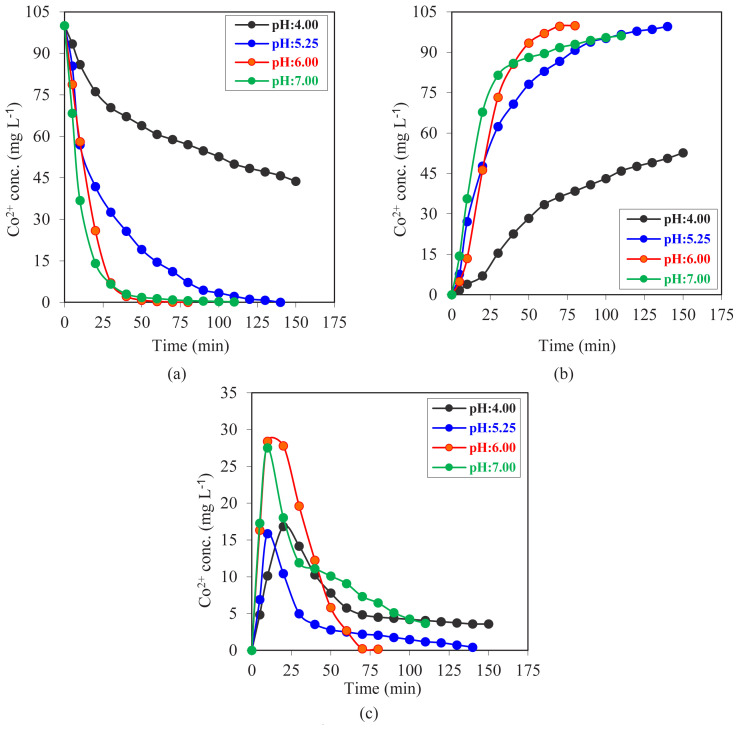
Concentrations of Co^2+^ ions versus time graphs for MDLM extraction studies of (a) donor, (b) acceptor, and (c) organic phase at different donor phase pH values (pH 4.00–7.00).

The effect of the donor phase pH on Co^2+^ ion transport kinetics in the MDLM system was studied in a series of experiments. The used pH values were 4.00, 5.25, 6.00, and 7.00. During the experiments, all other factors were held constant. The D2EHPA concentration was 0.023 mol L^−1^ in kerosene; the donor, acceptor, and organic phases were 100 mL each; and the donor phase Co^2+^ concentration was 100 mg L^−1^. The acceptor phase pH value was 1.50 and the pump speed was set to 20 rpm. Concentration changes with time for each individual phase are summarized in Figure 11. The graphs demonstrate that the pH of the donor phase has a direct effect on Co^2+^ ion transport between phases. Increasing the pH level of the donor phase brings fast transportation of ions between phases. On the other hand, over a pH of 6.00, accumulation in the organic phase increases and transfer between the phases is limited.

**Figure 12:**
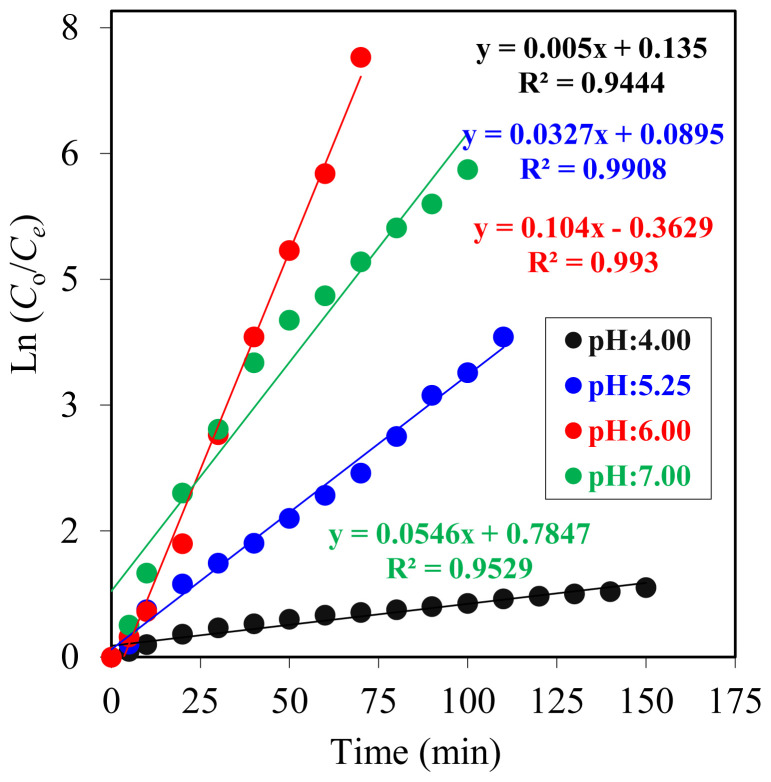
ln (*C*_o_/*C*_e_) versus time graphs for Co^2+^ ion MDLM extraction studies at different donor phase pH values (pH 3.00–7.00).

Donor phase pH value has a strong effect on reaction rate according to the experimental results. When the ln(*C*_o_/*C*_e_) versus time graph (Figure 12) is examined, it is clear that all individual datasets for different pH values ae straight lines with increasing slopes. At pH 4.00, the reaction in the donor phase was very slow, and in the acceptor phase, no reaction was observed. Bouranene at al. conducted research with a liquid emulsion membrane system for the removal of cobalt and lead ions from an aqueous medium. These researchers used D2EHPA as a carrier agent and investigated the effect of different experimental variables on the efficiency of the extraction system. They used different pH levels, ranging from 2.29 to 6.11, and according to their findings, low pH levels slowed the extraction because of cationic exchange difficulties at high acidity levels [31].

As illustrated in Figure 12, *R*^2^ values for all experiments were greater than 0.95, with the exception of pH 4.00 values. At pH 4.00, the reaction did not proceed as in other experiments, and this should be the reason for the inconsistency.

For each phase (donor, acceptor, and organic phase), concentration of Co^2+^ versus time graphs were drawn for different pH values between pH 4.00 and pH 7.00, as shown in Figure 11. According to the collected data, the donor phase pH of 7.00 had the highest transportation rate. Huang and Tsai used a liquid membrane technique, and they also used D2EHPA as a carrier agent dissolved in kerosene to extract Co^2+^ ions [32]. According to their provided results, with an increasing pH value in the feed phase, the transportation rate increased. With decreasing pH values, the transportation rate slowed, and the minimum transportation rate was monitored at pH 4.00. At pH 5.25 and 6.00, the transportation percentage was greater than 99.5%, indicating that Co^2+^ ions were successfully transported from one reactor to another. For pH 7.00 and pH 4.00, transportation occurred, but after a moderate reaction time, there were high amounts of Co^2+^ ions in the organic phase that could not be released in the acceptor phase.

In the course of the evaluation of kinetic parameters, as summarized in Table 3, smooth increases and decreases could be seen in *k*_1_, *k*_2_, $t_{m}^{max\cdot }$ and $R_{m}^{max.}$ values. As the pH of the donor phase increases, so do the *k*_1_ values. This result provides evidence of the rapid increase in *k*_1_ over pH 5.25. Furthermore, at pH 6.00 and 7.00, Co^2+^ ion accumulation is very high in the organic phase. Such a high extractant concentration in the liquid membrane phase is unfavorable for obtaining stable and controllable reaction conditions. Our results demonstrate that the most reliable kinetic factors can be provided by a donor phase pH value of 5.25.

Comparative data on the extraction of Co^2+^ ions with different membrane systems presented in the literature are given in Table 4.

Table 4 shows that Co^2+^ ions are extracted with a higher yield in the MDLM system than in other conventional systems under optimum conditions. In addition, the MDLM system has the advantage of being simple in terms of ease of installation compared to other systems and performing continuous extraction in a short time with less carrier ligand.

## 4. Conclusion

Co^2+^ ions were successfully extracted from an aqueous medium using the newly developed MDLM liquid membrane system. As variables, system parameters such as pH range, carrier agent concentration, and system temperature were considered and collected data were analyzed. The optimal parameters were found to be as follows: 1.5 and 5.25 pH values of the donor and acceptor phases, respectively; 0.023 mol L^−1^ D2EHPA concentration in the organic phase; and 25 °C system temperature. Under these conditions, the reaction had a high transport rate (99.56%) and the obtained kinetic data were reliable. Moreover, the reaction took place at the desired rate to allow the calculation of all kinetic data with high consistency. Considering the obtained findings, the transportation reaction was irreversible and of the first order. By using the data collected from experiments conducted at different temperatures, the activation energy of the reactions was calculated as 6.947 kcal mol^−1^. An activation energy greater than 10 kcal mol^−1^ indicates that the reaction between Co^2+^ ions and the carrier is kinetically controlled.

As a result, the MDLM system was shown to be an efficient system for extracting Co^2+^ ions with a high percentage of transport. This system resembles widely used BLM systems, but it has specific differences that make it preferable. The main advantages of the MDLM system are its ease of set-up, continuous transportation, high interaction area between the organic and water phases, high efficiency, and reasonable reaction durations.

## Figures and Tables

**Table 1:** Kinetic parameter comparison of Co^2+^ ion transportation via MDLM system with different carrier agent concentrations.

Conc. (mol L^−1^)	*k*_1_.10^2^ (min^−1^)	*k*_2_.10^2^ (min^−1^)	$t_{m}^{max\cdot }(\text{min})$	$R_{m}^{max.}(\text{mg }{\text{L}}^{-1})$	$J_{d}^{max}\cdot 10^{2}\;(\text{min})$	$J_{a}^{max}\cdot 10^{2}\;(\text{min})$
0.061	2.73	14.90	13.96	12.54	1.86	−1.86
0.045	3.54	12.00	14.44	17.72	2.12	−2.12
0.030	5.57	6.90	16.06	32.85	2.28	−2.28
0.023	4.46	9.50	15.00	24.02	2.28	−2.28
0.015	3.46	14.80	12.83	15.02	2.22	−2.22

**Table 2:** Kinetic parameter comparison of Co^2+^ ion transportation via MDLM system at different temperatures.

Temp (K)	*k*_1_.10^2^ (min^−1^)	*k*_2_.10^2^ (min^−1^)	$t_{m}^{max\cdot }(\text{min})$	$R_{m}^{max.}(\text{mg }{\text{L}}^{-1})$	$J_{d}^{max}\cdot 10^{2}\;(\text{min})$	$J_{a}^{max}\cdot 10^{2}\;(\text{min})$
293.15	2.23	6.32	25.46	19.98	1.26	−1.26
298.15	3.54	11.08	15.15	18.71	2.07	−2.07
303.15	6.14	9.19	13.22	29.66	2.73	−2.73
308.15	10.32	12.42	8.82	33.44	4.15	−4.15

**Table 3:** Kinetic parameter comparison of Co^2+^ ion transportation via MDLM system at different donor phase pH values.

pH	*k*_1_.10^2^ (min^−1^)	*k*_2_.10^2^ (min^−1^)	$t_{m}^{max\cdot }(\text{min})$	$R_{m}^{max.}(\text{mg }{\text{L}}^{-1})$	$J_{d}^{max}\cdot 10^{2}\;(\text{min})$	$J_{a}^{max}\cdot 10^{2}\;(\text{min})$
4.00	0.50	6.06	44.85	6.59	0.40	−0.40
5.25	3.30	11.07	15.58	17.83	1.97	−1.97
6.00	10.40	13.86	8.30	31.65	4.39	−4.39
7.00	5.60	6.94	16.01	32.92	2.28	−2.28

**Table 4:** Extractants used in Co^2+^ metal ion extraction via conventional membrane systems.

Method	Internal liquid	Type of extractant	Diluent	Surfactant	Stripping solution	Removal (%)	Ref.
LSM	H_2_SO_4_	HDEHP	Dodecane	SPAN 80	H_2_SO_4_	98.8	[31]
CSLM	HNO_3_	D2HEPA	-	-	EDTA	98.7	[33]
BLM	HCOOH	D2EHPA	Kerosene	-	H_2_SO_4_	50	[34]
ELM	NaSCN	CYANEX 923	Cyclohexane	SPAN 80	H_2_SO_4_	95	[35]
SLM	HCl	Triethanolamine	Cyclohexanone	-	NaOH	80	[36]
ELM	-	PC-88A	Kerosene	ECA 4360J	HCl	99	[37]
SLM	NaOH	LIX 860-I and D2EHPA	Kerosene	-	H_2_SO_4_	>90	[38]
MDLM	HNO_3_	D2EHPA	Kerosene	-	HNO_3_	99.56	This study
